# Genetic association study of *synphilin-1 *in idiopathic Parkinson's disease

**DOI:** 10.1186/1471-2350-9-19

**Published:** 2008-03-21

**Authors:** Ronny Myhre, Helge Klungland, Matthew J Farrer, Jan O Aasly

**Affiliations:** 1 Department of Laboratory Medicine, Children's and Women's Health, Norwegian University of Science and Technology, Trondheim, Norway; 2 Department of Neuroscience, Mayo Clinic College of Medicine, Jacksonville, Florida, USA; 3 Department of Neurology, St. Olav's Hospital, Trondheim, Norway

## Abstract

**Background:**

*Post-mortem *Lewy body and Lewy neuritic inclusions are a defining feature of Parkinson's disease (PD) and dementia with Lewy bodies (DLB). With the discovery of missense and multiplication mutations in the alpha-synuclein gene (*SNCA*) in familial parkinsonism, Lewy inclusions were found to stain intensely with antibodies raised against the protein. Yeast-two-hybrid studies identified synphilin-1 as an interacting partner of alpha-synuclein, and both proteins show co-immunolocalization in a subset of Lewy body inclusions. In the present study, we have investigated whether common variability in *synphilin-1*, including coding substitutions are genetically associated with disease pathogenesis.

**Methods:**

We screened the *synphilin-1 *gene for 11 single nucleotide polymorphisms (SNPs) in 300 affected subjects with idiopathic Parkinson's disease and 412 healthy controls. Six of these were rare variants including five previously identified amino acid substitutions that were chosen in a direct approach for association of rare disease causing mutations. An additional five highly heterozygous SNPs were chosen for an indirect association approach including haplotype analysis, based on the assumption that any disease causing mutations might be in linkage disequilibrium with the SNPs selected. We also genotyped a microsatellite marker (D5S2950) within intron 6 of the gene and five additional microsatellites clustered downstream of the 5p23.1-23.3 *synphilin-1 *locus. Genome-wide linkage analysis, in a number of independent studies, has previously highlighted suggestive linkage to PD in this region of chromosome 5.

**Results:**

Screening of previously known amino acid substitutions in the *synphilin-1 *gene, identified the C1861>T (R621C) substitution in four patients (chromosomes n = 600) and 10 control subjects (chromosomes n = 824), whereas the G2125>C (E706Q) substitution was detected in one patient and four control subject, suggesting both these substitutions are not associated with susceptibility to PD. Heterozygous non-synonymous T131>C (V44A) and synonymous C636>T (P212P) amino acid substitutions were each detected in only one patient with PD. Heterozygous C1134>T (L378L) synonymous substitutions were found in two patients with PD and one control subject. D5S2010 the most distal telomeric microsatellite marker genotyped,15.3 Mb from *synphilin-1*, was genetically associated with PD (p = 0.006, 27df) independently adjusted for multiple testing according to its high amount of alleles but not the total number of other markers investigated. Other flanking and intronic SNP and microsatellite markers showed no evidence for genetic association with disease.

**Conclusion:**

In this study rare *synphilin-1 *SNPs were assessed in a direct association approach to identify amino acid substitutions that might confer risk of PD in a homozygous or compound heterozygous state. We found none of these rare variations were associated with disease. In contrast to prior studies the frequency of the R621C substitution was not significantly different between PD and control subjects, neither were the V44A or E706Q substitutions. Similarly, our indirect study of more heterozygous SNPs, including both single marker and haplotype analyses, showed no significant association to PD. However, marginal association of microsatellite alleles with idiopathic PD, within the chromosome 5q21 region, indicates further studies are warranted.

## Background

Alpha-synuclein is an essential component of Lewy bodies, protein inclusion found *post-mortem *in affected subjects with synucleinopathies like PD[1], dementia with Lewy bodies (DLB)[2], and multiple system atrophy (MSA)[3,4]. Synphilin-1 was identified as an alpha-synuclein interacting protein *in vitro *in a yeast two-hybrid screen [5], and antibodies of Synphilin-1 have been found to stain Lewy bodies in brain cells in PD and DLB affected [6,7]. In a study performed by Wakabayashi and colleagues[6], Synphilin-1 was identified in the core of Lewy bodies with alpha-synuclein situated peripherally, suggesting a possible role of Synphilin-1 protein-protein interaction in Lewy body formation. Synphilin-1 has also been implicated in parkinsonism as a protein ubiquinated by Parkin for which loss of function results in juvenile or early-onset disease [8,9].

**Figure 1:**
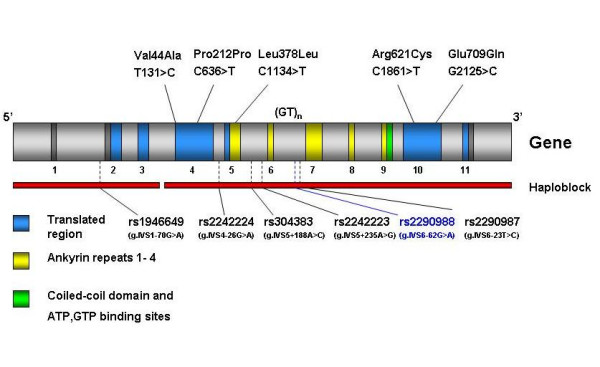
Organisation of the *synphilin-1 *gene with motifs as described by Engelender et al 2000 [10] and O'Farrell et al 2002 [11]. The SNPs genotyped are located on the figure relative to exons and motifs. Ankyrin-like motifs are highlighted in yellow. The coiled-coil motif with ATP and GTP binding domain are marked in green. Motifs involved in interactions with other proteins such as alpha-synuclein [29] and Parkin [9, 30] are not known.

S*ynphilin-1 *has several protein-protein interacting motifs, including Ankyrin repeats, a coiled coil domain and an ATP and GTP binding domain [10] (figure 1). By comparing the human and mouse *synphilin-1 *cDNA sequence it was confirmed that the regions containing these protein interacting motifs were among the most conserved together with a relatively long internal conserved region [11]. Amino acid substitutions in any of these domains may impair the protein's function [12].

The region of chromosome 5 in which the *synphilin-1 *locus resides has been implicated in PD is several independent genome wide screens [13-15] although a recent combined, pooled analysis was not supportive [16]. In this study we genotyped 11 SNPs in the *synphilin-1 *gene using rare variants in a direct association approach and a group of more informative heterozygous SNPs to assess by indirect means whether there was any evidence for genetic association with idiopathic PD. We also screened an internal microsatellite marker and five additional microsatellites downstream of the *synphilin-1 *5p23.1-23.3 locus [13-15].

## Methods

### PD affected and controls

A total of 305 affected have been clinically examined and followed longitudinally by one neurologist (JOA) at the outpatient clinics of three hospitals in Central Norway. Their diagnosis of PD was based on the presence of two or more of the cardinal symptoms including tremor, rigidity, bradykinesia and postural instability, with a positive response to levodopa, according to Gelb et al. 1999[17]. Five affected subjects did not fulfil the criteria and were omitted from the association study. They were diagnosed as PSP (n:2), MSA (n:1), CBD (n:1) and Pick's disease (n:1). All affected and controls were from Central Norway. The mean age at onset for the PD series was 58.9 ± 11.3 years; the mean age at study was 70.2 ± 10.7 (mean ± SD). Among the PD samples there were 113 females (37.7%) and 187 males (62.3%). Control subjects included 412 healthy individuals with age at examination 62.8 ± 10.6 years; (mean ± SD), 171 females (41.5%) and 241 males (58.5%). Four affected were found to have an additional relative included in the samples. No other family relationships were known within the affected samples. The Ethics Committees (IRB) of Central Norway and Mayo Clinic Jacksonville approved the study and informed consent was obtained from all participants.

### Marker selection

The strategy for selection of SNPs was bipartite. First, rare SNPs were selected in a direct approach to identify variants associated with PD. These included known variants of low frequency, of which five out of 6 result in non-synonymous or synonymous amino acid substitutions [10,12]. The low frequency variants were selected through scrutiny of previously published works by Engelender et al 2000 [10] and Marx et al 2003 [12] that identified some variants of interest to the study of idiopathic PD in *Synphilin-1*. Second, using an indirect approach for testing association within haplotype blocks, a group of informative SNPs (minor allele frequencies > 0.05) were selected from those available in the National Centre for Biotechnology Information database [18] (figure 1). In addition, a microsatellite, D5S2950, within *synphilin-1 *intron 6 characterized by Engelender and colleagues [10] was genotyped for association analysis. The selection of additional microsatellites, including D5S2115, D5S816, D5S414, D5S2116 and D5S2010, was based on the heterozygosity and location downstream of the *synphilin-1 *locus, spanning a region of chromosome 5 previously highlighted in genome-wide linkage analyses [13-15].

### Isolation of DNA and genotype analysis

DNA was isolated from 200 μl whole blood using QIAmp DNA Blood Mini Kit from Qiagen. All affected and control subjects were genotyped using SNaPshot^® ^multiplex kit and a subset confirmed by sequencing using Applied Biosystems Bigdye^® ^Terminator V3.1 cycle sequencing kit. DNA amplification was performed on an Applied Biosystems 2700 PCR machine, according to conditions recommended by the Applied Biosystems SNaPshot^® ^multiplex kit. Fluorescent SNPs and microsatellite products were subsequently sized and genotyped on an Applied Biosystems 3100 automated capillary machine. PCR conditions and primer sequences are available on request.

### Statistical analysis

Single marker and haplotype block association were estimated using Haploview version 3.2, estimating statistical significance by performing 10 000 permutations [19]. Haplotype blocks are defined according to Gabriel et al [20]. All chromosome marker locations were deduced from Ensembl gene [ID; ENSG00000064692][21]. Hardy-Weinberg equilibrium was estimated using PHARE [22]. Multiple marker association for microsatellites was estimated using CLUMP[23]. Differences in allele frequencies, OR, RR and chi-squared p-values were estimated using the Interactive Statistical Pages [24] by John C. Pezzullo PhD, of Georgetown University, Washington DC. Splice-site predictions of human *synphilin-1 *exon-intron sequence were performed using BDGP Splice Site Predictor set [25] for analysis of human sequence.

## Results

Using a direct approach, genotyping of known variants in the *synphilin-1*gene confirmed the presence of all the 6 variants in affected and/or control subjects (table 1).

**Table 1:** Genotype frequencies for single markers used in both direct and indirect association analysis obtained with the Haploview 3.2 software package. P-values and OR are for allelic analysis.

Exon/Intron	Nucleotide change	Chromosome location	Amino acid change	Genotypes Cases 11/12/22	Controls 11/12/22	MAF Cases/controls	Allele 1 vs. allele 2 *p*-value	OR (95% CI)
1	g.IVS1-70G>A	121 754 613	----	165/115/19	206/176/24	0.26/0.28	0.43	0.90 [0.71–1.15]
4	T131>C	121 786 462	Val44Ala	296/1/0	405/0/0	0.0/0.0	0.42	ND
4	C636>T	121 786 967	Pro212Pro	296/1/0	405/0/0	0.0/0.0	0.42	ND
5	g.IVS4-26G>A	121 788 920	----	153/115/32	97/181/34	0.30/0.30	0.91	0.98 [0.78–1.24]
5	C1134>T	121 789 001	Leu378Leu	295/2/0	404/1/0	0.0/0.0	0.58	2.73 [0.36–20.9]
6	g.IVS5+188A>C	121 789 313	----	133/118/42	173/181/53	0.34/0.35	0.78	0.97 [0.77–1.21]
6	g.IVS5+235A>G	121 789 360	----	151/115/33	196/183/32	0.30/0.30	0.95	1.01 [0.80–1.27]
7	g.IVS6-62G>A	121 804 161	----	291/6/0	397/8/0	0.01/0.01	1.00	1.02 [0.37–2.84]
7	g.IVS6-23T>C	121 804 200	----	154/118/27	202/179/31	0.29/0.29	0.86	0.98 [0.76–1.23]
10	C1861>T	121 814 302	Arg621Cys	293/4/0^†^	395/10/0	0.01/0.01	0.42	0.54 [0.18–1.64]
10	G2125>C	121 814 565	Glu709Gln	296/1/0	401/4/0	0.0/0.0	0.40	0.34 [0.05–2.27]

^†^One PSP affected (not accounted for here) was found to be homozygous for this variant. OR-Odds ratio. The SNPs used for indirect analysis were highly heterozygous; rs1946649, g.IVS1-70G>A (ObsHet; 0.41); rs2242224, g.IVS4-26G>A (ObsHet; 0.42); rs304383, g.IVS5+188A>C (ObsHet; 0.43); rs2242223, g.IVS5+235A>G (ObsHet; 0.42) and rs22900987, g.IVS6-23T>C (0.42).

The Arg621Cys (R621C)[12] substitution was identified as heterozygous in four affected and 10 control subjects. In addition, one of the two patient diagnosed with progressive supranuclear palsy (PSP) was homozygous for this relatively rare variant. Subsequent screening of an additional patient with PSP from the same geographical region did not reveal the variant. However, all patients with atypical parkinsonism were omitted from subsequent association analyses (table 1). A heterozygous Glu709Gln (E706Q) substitution was detected in one affected patient and four control subjects. A Val44Ala (V44A) non-synonymous substitution [10] in the first base of exon 4 was also detected in only one heterozygous female patient with a diagnosis of 'probable' PD with onset at 52 years. A synonymous substitution, C636>T, Pro212Pro[12] was identified in one heterozygous male patient with a diagnosis of 'atypical' PD, 60 years at onset, and in no control subjects. The base pair change C1134>T, leading to another synonymous substitution Leu378Leu [12] was observed in two patients and one control subject. A low frequency polymorphism g.IVS6-62G>A was found in 8 control subjects and 6 patients of both genders and at equal frequency (table 1).

**Figure 2:**
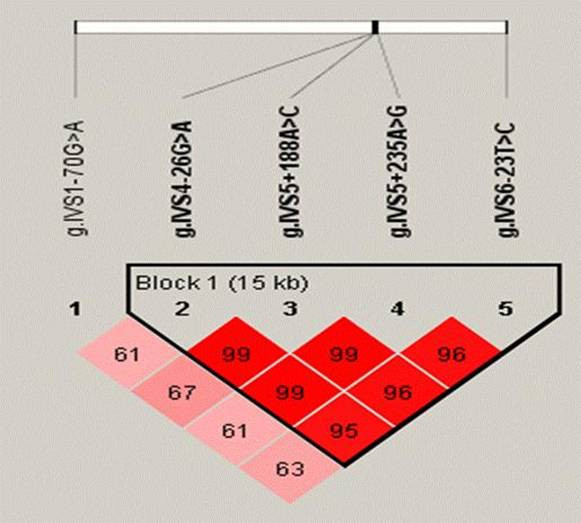
Software Haploview version 3.2 were used to estimate haplotype blocks. Haplotype blocks were defined according to Gabriel et al [20].

Highly heterozygous SNPs were used in an indirect association approach and tested for allele and haplotype association with disease. As presented in figure 1, all SNPs are situated in intronic regions in close proximity to exons. Single marker association (table 1) and haplotype analyses (table 2) showed no evidence of association between *synphilin-1 *and PD. The haplotype block structure observed (figure 2) corresponded with that identified for *synphilin-1 *in the HapMap project, using HapMap version 3.5.

**Table 2:** Haplotypes and haplotype frequencies in haploblock 1 as defined by default settings using Haploview version 3.2

**Haplotype block association**				
**Haplotype**	**Haploblock 1**	**MAF**	**Chi-square**	***p*-value**
GAAT	Haplotype A	0.35	0.13	0.72
GCAT	Haplotype B	0.34	0.18	0.67
AAGC	Haplotype C	0.28	0.01	0.93
AAGT	Haplotype D	0.02	0.35	0.55

Highly polymorphic markers were used for single marker (table 1) and haplotype association analysis. MAF-Minor allele frequency. P-values calculated using 10 000 permutations.

A downstream external microsatellite marker, D5S2010, showed significant association for PD (p = 0.006, 27 df). The remaining microsatellite markers, D5S2115, D5S816, D5S414 and D5S2116 showed no allelic differences when comparing PD with control subjects or in stratified analyses by diagnostic certainty, age or gender (data not shown). The microsatellite D5S2950 of *synphilin-1 *showed no overall association with disease (p = 0.48, 11 df) although some marginal allelic association was observed.

## Discussion

Despite functional research anticipating a role for *synphilin-1 *in Lewy body disorders, our results, and those of others performing association studies on family material and sporadic populations, do not provide support for a genetic association between PD and *synphilin-1 *[26-28]. Our results of rare SNPs were not significant and suggest that these genetic variants are unlikely to contribute to risk of PD although it must be noted that some of the SNP frequencies are very low and can not be conclusively excluded within the limits of the power of these analysis. A heterozygous intron-exon 4 splice acceptor site non-synonymous substitution T131>C (V44A), previously described by Engelender *et al *2000[10], was identified in a female patient with a 'probable' diagnosis of PD, with onset at 52 years, but was not found to segregate with PD in her affected brother. The substitution was absent in control subjects. Thus, while the variant may affect mRNA splicing it is unlikely to be a major contributor to disease risk.

Previously, Marx and colleagues [12] identified the C1861>T (R621C) substitution in two German patients with sporadic PD. In our sample from the Norwegian population, this variation was identified in four patients with PD (frequency 0.01) and 10 control subjects (frequency 0.02). Rather surprisingly, one patient with PSP was homozygous for the R621C substitution. However, PSP is a tauopathy in which *synphilin-1 *is unlikely to play a role, and no other PSP patients were identified with the mutation. Our findings suggest C1861>T (R621C) is only a polymorphism. The case-control distribution of other SNPs was approximately equal between groups. No statistical difference was found for single marker or haplotype block associations, adjusting for age and gender. We examined five external microsatellite markers, including D5S816 marker which has previously been implicated in PD through a genome wide analysis of affected sib-pairs [14]. In our population there were no significant differences between affected, controls and diagnosis for any of the external markers except D5S2010 (p = 0.006) for which neighbouring genetic variability may warrant further analysis. Interesting and surprisingly, the D5S2010 marker is more or less directly linked to CENTD3 an effector of PI3K the gate controller of autophagy, cell survival and cell death that complexes with SH3KBP1 on Xp22.1-p21.3.

## Conclusion

We found no evidence for association between genetic variability in *synphilin-1 *and PD. There were no statistical differences in allele or genotype frequencies between PD or control subjects, for common or rare variants within the *synphlin-1 *gene. Although synphilin-1 appears to be present within Lewy bodies, may interact with alpha-synuclein, and may be a substrate of Parkin, any genetic evidence for its involvement in disease, either through sequencing and the identification of rare coding substitutions, association or linkage, is undetected in this Norwegian population study and remains to be confirmed.

## Competing interests

The author(s) declare that they have no competing interests.

## Authors' contributions

RM, writing of manuscript, laboratory work, statistical analysis. HK, contribution to conception and design in Norway. MJF, writing of manuscript, contribution to conception and design at Mayo clinic Jacksonville. JOA, neurologist assessing the diagnosis of the affected, contribution to conception and design in Norway

## Pre-publication history

The pre-publication history for this paper can be accessed here:


